# Effect of knee sleeves on joint angle variability during gait in older adults: a principal component analysis

**DOI:** 10.3389/fbioe.2025.1525174

**Published:** 2025-05-20

**Authors:** Wakako Tsuchida, Takuma Inai, Shoma Kudo, Yoshiyuki Kobayashi, Masahiro Fujimoto

**Affiliations:** ^1^ Integrated Research Center for Self-Care Technology, National Institute of Advanced Industrial Science and Technology (AIST), Takamatsu, Kagawa, Japan; ^2^ Health and Medical Research Institute, National Institute of Advanced Industrial Science and Technology (AIST), Takamatsu, Kagawa, Japan; ^3^ Research Institute on Human and Societal Augmentation, National Institute of Advanced Industrial Science and Technology (AIST), Kashiwa, Chiba, Japan; ^4^ Integrated Research Center for Self-Care Technology, National Institute of Advanced Industrial Science and Technology (AIST), Kashiwa, Chiba, Japan

**Keywords:** walking, knee sleeve, joint angle variability, principal component analysis, older adults

## Abstract

**Introduction:**

As the global elderly population increases, maintaining walking ability and minimizing fall risk among older adults is crucial for their health and wellbeing. Knee sleeves are commonly utilized in geriatric sports and rehabilitation to stabilize knee joint movement and enhance gait stability. However, their effects on joint kinematics during walking in healthy older adults, particularly on joint angle variability—a factor associated with fall risk—remain inadequately explored. This study aimed to investigate the influence of knee sleeves on joint angle variability during gait in healthy older adults.

**Methods:**

Principal component analysis was performed on 16 healthy older adults residing in the community, utilizing 3D spatiotemporal data of the participants’ time-normalized lower limb joint angles throughout the complete gait cycle. The analysis involved a 64 × 1818 input matrix, accounting for 16 participants, two conditions (control and knee sleeves), two walking speeds (normal and fast), three angles, three axes, 101 time points, and two parameters (average and variability). Kinematic waveforms were reconstructed based on the statistical findings to identify notable differences in joint angle variabilities between the conditions.

**Results and discussion:**

The outcomes revealed reduced variability in knee and ankle joint angles on the sagittal plane when walking with knee sleeves compared to walking without them. Conversely, an increased variability in hip, knee, and ankle joint angles was observed on the sagittal plane when walking at a fast speed compared to a normal speed. These results suggest that knee sleeves may reduce variability in knee and ankle joint angles during walking in older adults, potentially lowering the risk of falls. This effect appears particularly beneficial during fast-paced walking, where joint angle fluctuations are more pronounced than during normal-speed walking. These findings offer quantitative evidence for the effectiveness of knee sleeves in enhancing walking performance in healthy older adults.

## 1 Introduction

As the global elderly population grows, developing effective strategies to prevent falls is crucial due to the significant impact of the health and wellbeing of older adults, as expressed by the WHO Ageing and Health Unit (2008), and more recently by Xu et al. (2022). Knee sleeves are commonly utilized to offer compression, support, and warmth to the knee joint. They are not only used in geriatric sports and rehabilitation settings for injury or disability but also by healthy older adults to prevent injury and enhance comfort during daily activities (Beaudreuil et al., 2009; Mohd Sharif et al., 2017; Dzidotor et al., 2024). These benefits are expected to stabilize joint movements and improve gait stability. Previous research has indicated that knee sleeves may enhance functional and performance safety by reducing joint pain and minimizing excessive knee loading during sports activities (Beaudreuil et al., 2009; Collins et al., 2011; Schween et al., 2015; Nyland et al., 2015; Ko et al., 2017; Mohd Sharif et al., 2017; Dzidotor et al., 2024). However, the observed enhancements in gait and functional aspects are predominantly limited to individuals with pathological knee conditions, such as osteoarthritis, and the effect of knee sleeves on healthy older adults is still uncertain. Therefore, it remains unclear whether knee sleeves would provide joint stability and improve walking performance in healthy older populations.

Recent research indicates that gait variability is commonly used to evaluate instability and predict the risk of falls in older adults, highlighting its significance in geriatric health evaluations (Herman et al., 2005; Moe-Nilssen and Helbostad, 2005; Brach et al., 2005; Brach et al., 2007; Lord et al., 2011; Al Abiad et al., 2023; Chan et al., 2023; Cronström et al., 2022; Commandeur et al., 2024; Osman et al., 2023). However, to our knowledge, no prior study has explored the effects of knee sleeves on gait variability in healthy individuals. Detailed investigation of the effects of using knee sleeves on gait characteristics, including the variability in joint kinematics during walking, could enhance our understanding of how knee sleeves influence gait function and its underlying mechanisms.

Several studies have investigated the effects of knee sleeves on various gait variables, such as walking speed and lower limb joint angles (Collins et al., 2011; Schween et al., 2015; Ko et al., 2017). However, these conventional methods often focus solely on specific variables at discrete time points, potentially overlooking crucial information in significant portions of unanalyzed data, as previously highlighted (Nigg et al., 2012). Principal component analysis (PCA) has been utilized to identify movement characteristics across different groups and conditions by comprehensively analyzing waveforms from entire time-series data (Deluzio and Astephen, 2007; Maurer et al., 2012; Nigg et al., 2012; Kobayashi et al., 2014; Kobayashi et al., 2016; Nakajima et al., 2018; Kobayashi and Ogata, 2018; Hida et al., 2021; Tsuchida et al., 2022; Inai et al., 2023). In particular, kinematic waveforms showing distinct differences between groups were reconstructed using principal component vectors (PCVs). This method was used to characterize variations in the movements of recent fallers and non-fallers and to establish the relationship between the risk of falling and joint kinematic variability of the lower limbs during walking (Kobayashi et al., 2014). Therefore, using a PCA-based approach can enhance our understanding of joint kinematics with knee sleeves throughout the gait cycle.

Given that walking at different speeds can impose varying demands on postural control and gait stability (Jordan et al., 2007; Johansson et al., 2016; Padulo et al., 2023), it is necessary to analyze gait variability across both normal and fast walking conditions. Several studies have demonstrated that gait variability increases at higher speeds, potentially raising fall risk (Jordan et al., 2007; Johansson et al., 2016; Padulo et al., 2023; Quach et al., 2011). Thus, investigating whether knee sleeves provide stability benefits under different conditions is crucial for understanding their effectiveness in real-world scenarios.

This study aimed to examine the effects of knee sleeves on joint angle variability throughout the gait cycle in healthy older adults using PCA. Participants walked at both a comfortable and a faster pace to evaluate the extent to which knee sleeves influence gait variability.

## 2 Methods

### 2.1 Participants

Sixteen healthy community-dwelling older adults participated in this study (eight males and eight females, age: 71.8 ± 3.4 years, height: 1.59 ± 0.09 m, body mass: 55.5 ± 8.5 kg, body mass index: 21.8 ± 1.7 kg/m^2^). The sample size was determined using G*Power software (version 3.1.9.6, Heinrich Heine University Dusseldorf, Germany). The *a priori* power analysis for the repeated-measures design indicated that a sample size of 16 would be sufficient to demonstrate a significant result with an alpha level of 0.05, a power of 0.8, and an effect size of 0.78, as estimated based on a previous study with a comparable design examining the effect of walking speed on gait cycle variability (Jordan et al., 2007). All participants were in good general health, with no history of knee surgery, musculoskeletal disorders, or neurological conditions. They were excluded if they needed assistive devices, had undergone surgery for trauma or orthopedic conditions, had neurological disorders, or were professional athletes. All participants were able to walk independently, had normal or corrected vision, and were free of any known diseases. They maintained a regular diet and refrained from vigorous activity before the experiment. The study adhered to the Declaration of Helsinki and received approval from the AIST Ethics Committee in Japan (IRB number: hi2023-534). All participants provided written informed consent.

### 2.2 Instrumentation and data collection

Three-dimensional (3D) positional data were collected during walking by employing reflective markers and a 10-camera motion capture system (MAC3D, Motion Analysis Corporation, Santa Rosa, CA, United States) operating at a sampling frequency of 200 Hz. Each participant’s bony landmarks were marked with a total of 57 infrared reflective markers following the Visual 3D software guidelines (HAS-Motion Inc., Kingston, ON, Canada). Prior to the walking trials, the marker positions were recorded while standing stationary.

### 2.3 Protocol

Gait measurements were conducted in a room with a straight 15-m path for participants to walk on. Participants walked barefoot back and forth along the 15-m straight path five times at two different speeds (normal and fast), both with and without knee sleeves (knee sleeves and control). They were instructed to walk at a comfortable speed and then as fast as possible in the normal- and fast-speed conditions. Knee sleeves (Actcyc Walk [ACT-80], Kagawa Seamless Inc., Kagawa, Japan) were worn on both knees during the sleeve condition, while the participants walked without them during the control condition. A knee sleeve with a standard wraparound design, without a hinge, was chosen to accommodate regular use by the participants. Given the potential effect of fast walking on subsequent comfortable walking speeds and patterns (Zhang et al., 2022), the normal speed condition preceded the fast speed condition. The sequence of control and knee-sleeve conditions was counterbalanced among the participants.

To ensure data consistency, all participants walked barefoot at all times. All trials were conducted in a controlled indoor environment with stable lighting and minimal external disturbances. The walking surface was a low-pile carpet, providing uniform and controlled conditions. Additionally, all participants wore the same type of clothing during the experiment (i.e., sleeveless shirt and spats), provided by the experimenter. Clothing sizes ranged from extra small to extra-large and were selected by the participants. The experimental setup remained unchanged throughout the study.

### 2.4 Data analysis

The marker trajectory data were digitally filtered using a zero-lag, fourth-order, low-pass Butterworth filter with a cutoff frequency of 10 Hz (van den Bogert and de Koning, 1996). Analysis was conducted on the lower limb joints (hip, knee, and ankle) associated with the risk of falling during walking (Kobayashi et al., 2014). Hip, knee, and ankle joint angles throughout a single gait cycle were calculated for the x-axis, y-axis, and z-axis using a Cardan sequence of rotations (X-Y-Z) based on the measured trajectories in each trial (Kobayashi et al., 2014; Kobayashi et al., 2016; Kobayashi and Ogata, 2018; Hida et al., 2021; Tsuchida et al., 2022). Means values and within-participant variability (coefficient of variation) of walking speed, step length, stride length, cadence, step time, and stride time were determined to elucidate gait characteristics. The coefficient of variation was utilized to validate the relative variability of the spatiotemporal gait parameters of gait. The procedures of low-pass filtering, variable computations, and time normalization were performed using Visual 3D software (HAS-Motion Inc., Kingston, ON, Canada).

### 2.5 PCA

In this study, the following six steps were used to perform the PCA. (1) The lower-limb joint angles were time-normalized using the gait cycle duration determined by two consecutive heel-strikes of the same leg. They were then divided into 101 time points ranging from 0% to 100% (Jordan et al., 2007; Padulo et al., 2023). The average and standard deviation (SD) within each time point were computed across five trials for each leg of every participant. (2) Mean centering was applied to all 1818 variables, which included averages and SDs for the 101 time points, two parameters (average and SD), and three angles in three axes, using the z-score.

$$z_{t}=\left(X_{t}-\mu_{t}\right)/\sigma_{t}$$

where $z_{t}$ is the z-score for the parameter *t*, $X_{t}$ represents the raw data of the parameter *t*, $\mu_{t}$ denotes the mean of the parameter *t* for the participant, and $\sigma_{t}$ is SD of the parameter *t*. (3) Matrices comprising 64 data points (16 participants, two conditions, and two walking speeds) across 1818 variables were constructed. (4) PCVs were extracted based on Kaiser’s criterion (eigenvalue >1) and a cumulative variance threshold of 80% (Jolliffe, 2002). (5) Subsequent statistical analyses were performed to ascertain the primary effects of knee sleeve condition and walking speed on the joint kinematic characteristics as indicated by the PCVs. (6) Joint kinematic average and SD waveforms were reconstructed using PCVs in cases where significant differences in the principal component scores (PCSs) were detected between normal and fast walking speeds, as well as between control and knee sleeve conditions, employing the methodologies outlined by Kobayashi et al. (2014), Tsuchida et al. (2022), and Deluzio and Astephen (2007). Three SD values were added to and subtracted from the grand-mean of each of the 1818 data points based on the polarity of the PCSs to highlight differences between conditions and speeds. In instances where significant differences were observed in multiple PCSs, joint kinematic waveforms were reconstructed based on the relative weight ratio of each PCV. Increase or decrease in the reconstructed SD waveforms, emphasizing either walking with knee sleeves or at a fast-walking speed, respectively, were calculated as the percentage of deviation for each condition relative to the grand mean waveform. Principal component loadings (PCLs) were calculated to identify the variables that significantly affected the principal components. The PCLs reflect the correlations between the original variables and the principal components, with a correlation coefficient of 0.7 or higher considered indicative of a strong correlation (Rojas-Valverde et al., 2020).

### 2.6 Statistical analysis

A two-way repeated-measures analysis of variance (ANOVA) with condition (control and knee sleeves) and speed (normal and fast) was utilized to investigate the main and interaction effects. The index of effect size (d for pairwise comparison; partial eta-squared for ANOVA) was presented as p-values. Small, medium, and large effects were defined as 0.01–0.06, 0.06–0.14, and >0.14, respectively (Cohen, 1992). A relative weight analysis was conducted to assess the relative weight ratios of each PCV. The statistical analyses were carried out using the SPSS statistical software package (IBM SPSS Statistics Version 29, SPSS Inc., Chicago, IL, United States) and R language 4.3.0 (R Core Team, Vienna, Austria). Statistical significance was established at p < 0.05 for all comparisons.

## 3 Results

PCA revealed that 13 extracted PCVs explained over 80% of the joint movement patterns. A scree plot illustrating the variance explained by each principal component is shown in Supplementary Figure S1. The variances, means, and SDs of the PCSs for each group are detailed in Table 1. There was no significant interaction between the conditions (with and without knee sleeves) and speeds (normal and fast) concerning the PCVs. A significant main effect of the condition was noted for PCV 1 (p = 0.049, η^2^ = 0.23). Significant main effects of speed were observed for PCVs 1, 3, 4, 6, 7, 8, and 10 (p ≤ 0.044, $\eta_{p}^{2}$ ≥ 0.24).

**TABLE 1:** Results of main principal component analysis.

Variable	Group	Statistics	PCV1			PCV2			PCV3			PCV4			PCV5		
Explained variance (%)			17.92			13.80			11.55			7.21			6.69		
Cumulative (%)			17.92			31.72			43.28			50.49			57.18		
Control (mean ± SD)			0.12 ± 1.04			−0.01 ± 1.02			0.07 ± 1.00			0.01 ± 1.00			0.11 ± 1.01		
Knee sleeve (mean ± SD)			−0.12 ± 0.85			0.01 ± 0.96			−0.07 ± 0.94			−0.01 ± 0.97			−0.11 ± 0.90		
Normal (mean ± SD)			−0.24 ± 0.90			0.00 ± 0.96			−0.18 ± 0.91			−0.14 ± 0.94			0.22 ± 0.97		
Fast (mean ± SD)			0.24 ± 0.99			0.00 ± 1.02			0.18 ± 1.03			0.14 ± 1.02			−0.22 ± 0.94		
Factor	Condition	*p*-value	0.05*			0.78			0.08			0.76			0.77		
		Partial *η*2	0.23			0.01			0.19			0.01			0.01		
	Speed	*p*-value	0.02*			0.92			0.03*			0.04*			0.24		
		Partial *η*2	0.33			0.00			0.29			0.24			0.09		
Interaction		*p*-value	0.10			0.48			0.71			0.50			0.87		
		Partial *η*2	0.17			0.03			0.01			0.03			0.00		

Variable	Group	Statistics	PCV6			PCV7			PCV8			PCV9			PCV10		
Explained variance (%)			5.34			4.23			3.64			3.05			2.34		
Cumulative (%)			62.52			66.75			70.39			73.44			75.78		
Control (mean ± SD)			−0.08 ± 0.96			0.00 ± 1.02			0.08 ± 0.99			0.08 ± 0.90			−0.21 ± 0.86		
Knee sleeve (mean ± SD)			0.08 ± 0.93			0.00 ± 0.88			−0.08 ± 0.92			−0.08 ± 1.06			0.21 ± 0.97		
Normal (mean ± SD)			0.27 ± 0.94			−0.27 ± 0.94			0.22 ± 0.87			0.02 ± 0.96			0.29 ± 0.82		
Fast (mean ± SD)			−0.27 ± 0.95			0.27 ± 0.95			−0.22 ± 1.05			−0.02 ± 1.00			−0.29 ± 1.01		
Factor	Condition	*p*-value	0.05			0.98			0.12			0.21			0.06		
		Partial *η*2	0.22			0.00			0.15			0.10			0.21		
	Speed	*p*-value	<0.001*			<0.001*			0.04*			0.85			0.006*		
		Partial *η*2	0.63			0.54			0.26			0.00			0.40		
Interaction		*p*-value	0.11			0.29			0.59			0.15			0.78		
		Partial *η*2	0.16			0.08			0.02			0.14			0.01		

Variable	Group	Statistics	PCV11			PCV12			PCV13		
Explained variance (%)			2.14			1.97			1.72		
Cumulative (%)			77.92			79.89			81.61		
Control (mean ± SD)			0.09 ± 1.02			0.05 ± 1.01			0.02 ± 1.10		
Knee sleeve (mean ± SD)			−0.09 ± 1.01			−0.05 ± 0.93			−0.02 ± 0.83		
Normal (mean ± SD)			−0.24 ± 1.01			−0.01 ± 1.07			0.09 ± 1.06		
Fast (mean ± SD)			0.24 ± 1.03			0.01 ± 0.87			−0.09 ± 0.86		
Factor	Condition	*p*-value	0.20			0.59			0.85		
		Partial *η*2	0.11			0.02			0.00		
	Speed	*p*-value	0.55			0.96			0.42		
		Partial *η*2	0.02			0.00			0.04		
Interaction		*p*-value	0.29			0.33			0.11		
		Partial *η*2	0.08			0.06			0.16		

The “*” symbol indicates *p* < 0.05.

**FIGURE 1:**
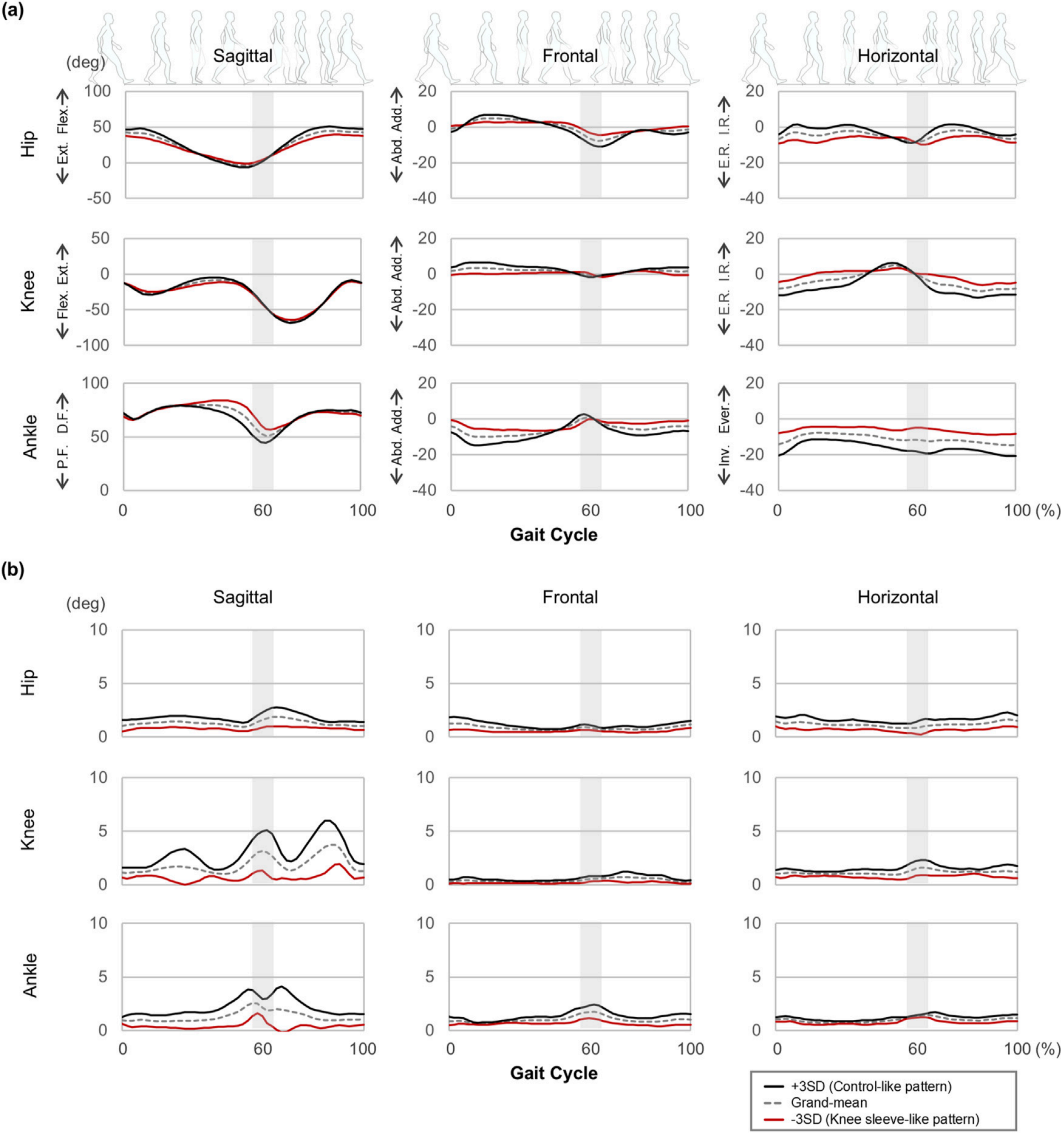
Waveforms of **(a)** central tendency (average) and **(b)** variability (standard deviation: SD) reconstructed from the PCSs of PCV 1. Abbreviations in the graph are defined as follows: Post.: Posterior Tilt, Ant.: Anterior Tilt, Flex.: Flexion, Ext.: Extension, D.F.: Dorsi-flexion, P.F.: Plantar flexion, Hike.: Pelvic hike, Drop.: Pelvic drop, Add.: Adduction, Abd.: Abduction, I.R.: Internal Rotation, E.R.: External Rotation, Ever.: Eversion, Inv.: Inversion. The gray highlighted area represents the moment of toe-off, marking the transition from the stance phase to the swing phase. The width of this area reflects that the stance and swing phases were not separated during the time-normalization procedure.

**FIGURE 2:**
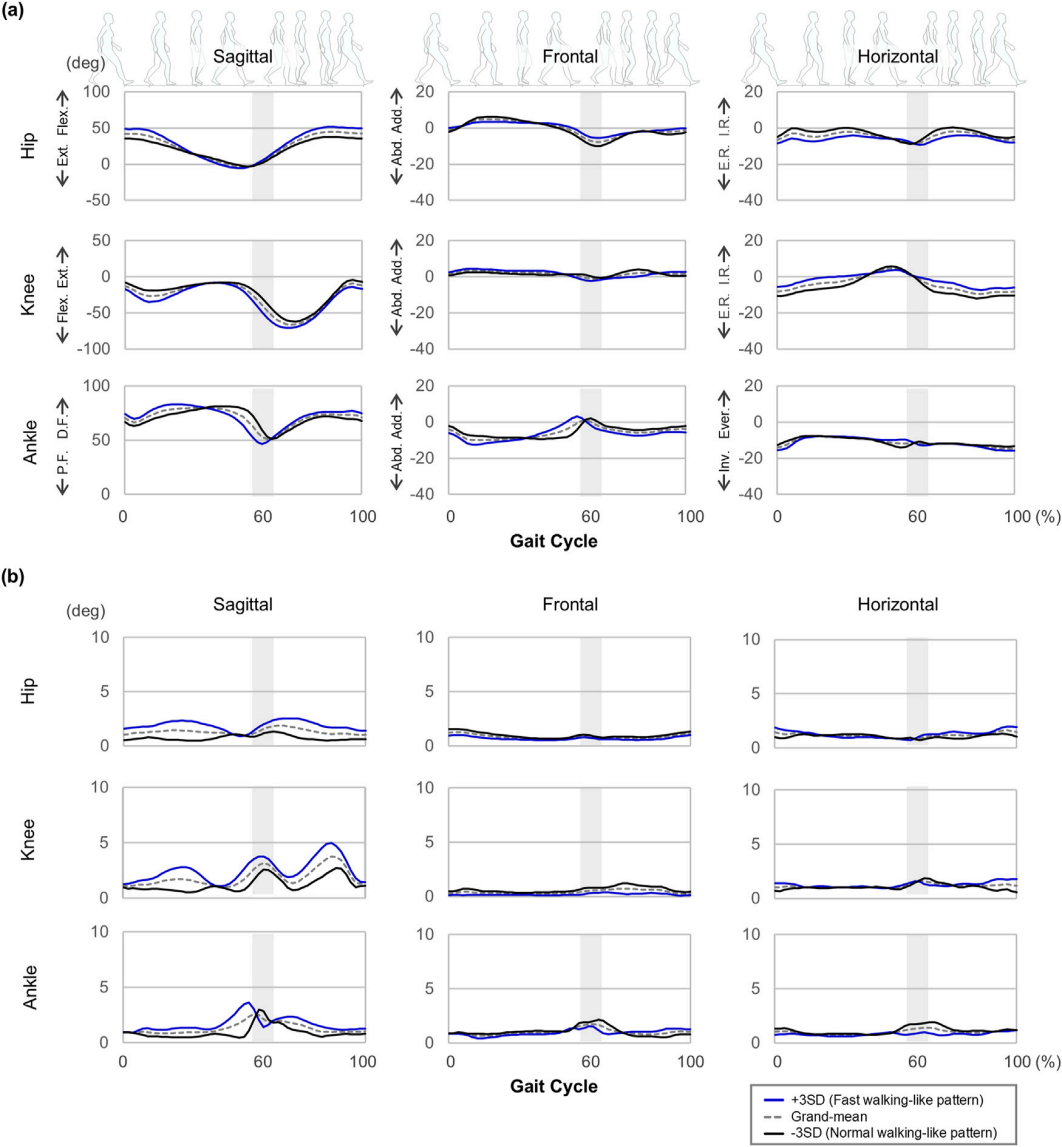
Waveforms of **(a)** central tendency (average) and **(b)** variability (standard deviation: SD) reconstructed from the PCSs of PCV 1, 3, 4, 6, 7, 8, and 10. Abbreviations in the graph are defined as follows: Post.: Posterior Tilt, Ant.: Anterior Tilt, Flex.: Flexion, Ext.: Extension, D.F.: Dorsi-flexion, P.F.: Plantar flexion, Hike.: Pelvic hike, Drop.: Pelvic drop, Add.: Adduction, Abd.: Abduction, I.R.: Internal Rotation, E.R.: External Rotation, Ever.: Eversion, Inv.: Inversion. The gray highlighted area represents the moment of toe-off, marking the transition from the stance phase to the swing phase. The width of this area reflects that the stance and swing phases were not separated during the time-normalization procedure.

Given these significant differences, PCV one was used to reconstruct the joint kinematic waveforms (average and SD) on the sagittal, frontal, and horizontal planes, highlighting the distinctions between the conditions (Figure 1). Similarly, PCVs 1, 3, 4, 6, 7, 8, and 10 were used to illustrate the differences of speed (Figure 2).

Focusing on within-participant SD, the reconstructed waveforms, while emphasizing walking with knee sleeves, showed the following deviations from the grand-mean waveform: in the sagittal plane, joint angle variability was reduced by up to 53.5% in the hip, 100.5% in the knee, and 108.1% in the ankle. In the frontal plane, reductions were observed by up to 47.5% in the hip, 49.1% in the knee, and 48.7% in the ankle. In the horizontal plane, variability decreased by up to 48.0% in the hip, 41.7% in the knee, and 25.0% in the ankle (Figure 1b).

When walking at a fast speed, the reconstructed waveforms revealed the following deviations from the grand-mean waveform: in the sagittal plane, joint angle variability increased by up to 72.8% in the hip, 82.7% in the knee, and 84.5% in the ankle. In the frontal plane, increases were observed by up to 33.9% in the hip, 49.9% in the knee, and 41.9% in the ankle. In the horizontal plane, variability increased by up to 32.6% in the hip, 40.4% in the knee, and 43.5% in the ankle (Figure 2b).

**FIGURE 3:**
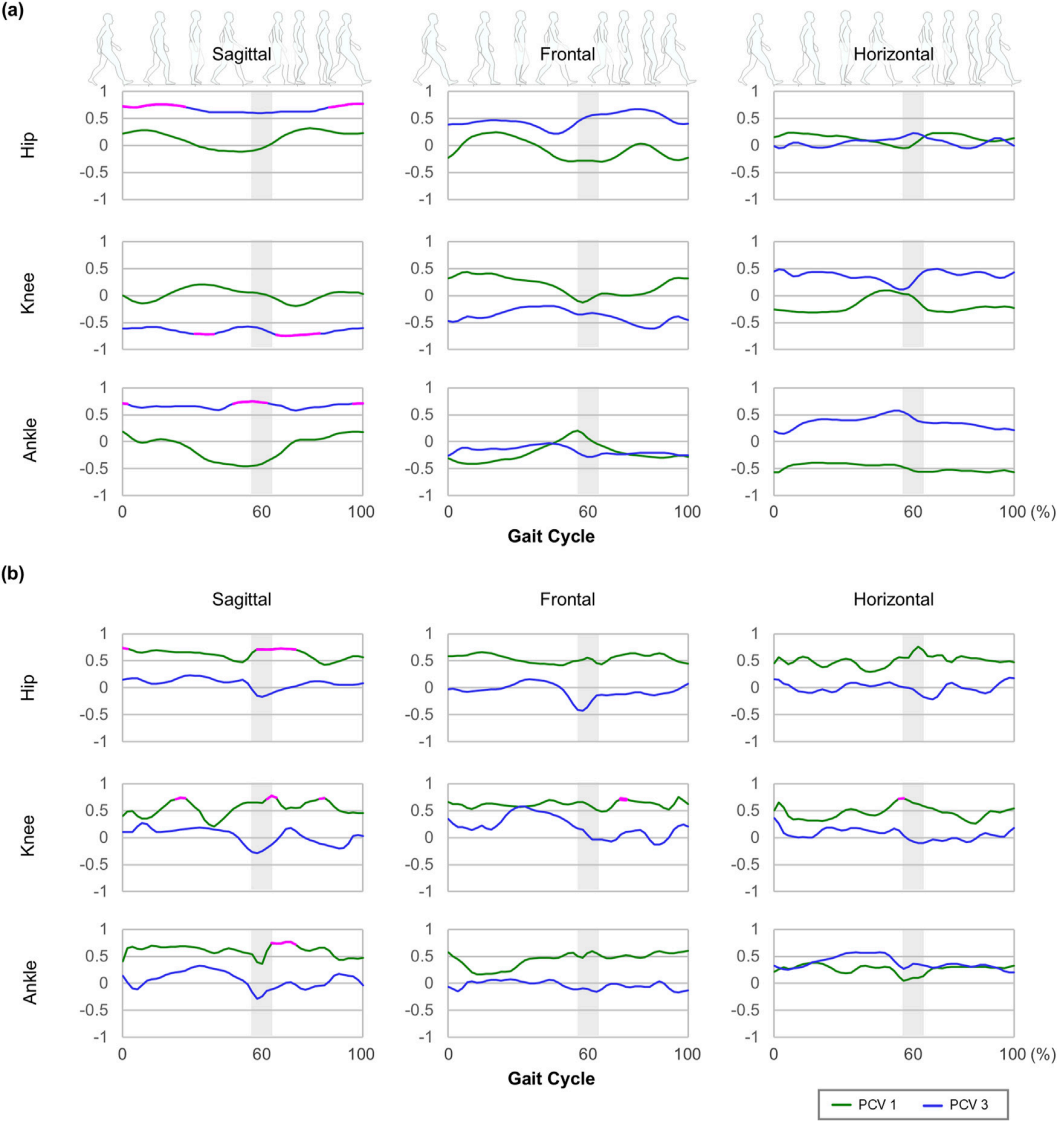
Waveforms of principal component loadings of PCV 1 and 3: **(a)** central tendency (average) and **(b)** variability (standard deviation: SD). The magenta solid line indicates that the absolute value of the principal component loading is greater than 0.7.

Among the PCLs for PCV 1, 3, 4, 6, 7, 8, and 10, strong correlations were observed during several key phases in PCV one and 3 (Figure 3). Strong correlations for within-participant variability were confirmed during the early stance and early swing phases in the sagittal plane of the hip joint, mid-stance, and early and mid-swing phases in the sagittal plane, mid-swing phase in the frontal plane, double-limb support phase in the horizontal plane of the knee joint, and early swing phase in the sagittal plane of the ankle joint (Figure 3b).

For the average and variability of spatiotemporal gait parameters, no significant interactions between speed and condition were observed (Table 2). Significant main effects of speed were observed on various gait parameters, including walking speed, step length, stride length, cadence, step time, and stride time (p ≤ 0.001). Significant effects of speed were also noted for within-participant variability in walking speed (p = 0.013) and stride length (p = 0.048). There were no significant effects of the conditions on any of the parameters.

**TABLE 2:** Results of the central tendency and the variability (coefficient of variation) of the spatiotemporal parameters.

(a) Central tendency of gait parameters												
Variable	Control (mean±SD)			Knee sleeve (mean±SD)			Normal (mean±SD)			Fast (mean±SD)		
Walking speed (m/s)	1.51 ± 0.26			1.52 ± 0.27			1.32 ± 0.15			1.71 ± 0.21		
Step length (m)	0.67 ± 0.07			0.67 ± 0.07			0.64 ± 0.06			0.70 ± 0.08		
Stride length (m)	1.34 ± 0.15			1.33 ± 0.15			1.27 ± 0.11			1.40 ± 0.15		
Cadence (steps/min)	134.8 ± 18.6			136.9 ± 19.0			124.4 ± 10.5			147.3 ± 18.29		
Step time (s)	0.45 ± 0.06			0.45 ± 0.06			0.49 ± 0.04			0.41 ± 0.05		
Stride time (s)	0.91 ± 0.12			0.89 ± 0.11			0.97 ± 0.09			0.83 ± 0.10		

	Factor					
	Condition		Speed		Interaction	
Variable	*p*-value	Partial η^2^	*p*-value	Partial η^2^	*p*-value	Partial η^2^
Walking speed (m/s)	0.18	0.12	<0.001*	0.84	0.41	0.05
Step length (m)	0.41	0.05	<0.001*	0.62	0.80	0.01
Stride length (m)	0.46	0.04	<0.001*	0.61	0.77	0.01
Cadence (steps/min)	0.14	0.14	<0.001*	0.67	0.48	0.03
Step time (s)	0.16	0.13	<0.001*	0.74	0.63	0.02
Stride time (s)	0.13	0.14	<0.001*	0.74	0.64	0.02

(b) Variabilities of gait parameters												
Variable	Control (mean±SD)			Knee sleeve (mean±SD)			Normal (mean±SD)			Fast (mean±SD)		
Walking speed (%)	2.66 ± 0.71			2.56 ± 1.21			2.25 ± 0.86			2.97 ± 1.06		
Step length (%)	2.75 ± 0.85			2.67 ± 1.21			2.50 ± 1.16			2.92 ± 0.90		
Stride length (%)	2.23 ± 0.71			2.20 ± 1.06			2.00 ± 0.97			2.44 ± 0.81		
Cadence (%)	1.74 ± 0.55			1.77 ± 0.67			1.63 ± 0.54			1.89 ± 0.68		
Step time (%)	2.50 ± 0.66			2.50 ± 0.86			2.43 ± 0.67			2.57 ± 0.85		
Stride time (%)	1.74 ± 0.55			1.77 ± 0.67			1.63 ± 0.54			1.88 ± 0.68		

	Factor				Interaction	
	Condition		Speed			
Variable	*p*-value	Partial η^2^	*p*-value	Partial η^2^	*p*-value	Partial η^2^
Walking speed (%)	0.60	0.02	0.02*	0.35	0.65	0.01
Step length (%)	0.66	0.01	0.09	0.18	0.23	0.10
Stride length (%)	0.82	0.00	0.05*	0.24	0.15	0.14
Cadence (%)	0.74	0.01	0.14	0.14	0.96	0.00
Step time (%)	0.65	0.02	0.65	0.01	0.13	0.15
Stride time (%)	0.77	0.01	0.14	0.14	0.99	0.00

The “*” symbol indicates p < 0.05.

## 4 Discussion

This study aimed to compare the variability in joint angles during the entire gait cycle with and without knee sleeves in older individuals. PCA was performed on the time-normalized average and SD of the lower limb joint angles. A significant main effect of condition (with/without knee sleeves) was observed for PCV 1, and significant main effects of walking speed were identified for seven PCVs: 1, 3, 4, 6, 7, 8, and 10, which were subsequently utilized to reconstruct the kinematic waveforms of the joint angles. The reconstructed waveforms of the within-participant SD, particularly during fast-paced walking, indicated a greater variability in the hip, knee, and ankle joint angles in the sagittal plane (over 72%). When focusing on joint angle characteristics with knee sleeves, a reduced variability was observed in the knee and ankle joint angles in the sagittal plane (over 100%). These results suggest that walking at a fast pace increases the variability of the lower limb joint angles in the sagittal plane, while knee sleeves decrease the variability in the knee and ankle joint angles during walking.

The larger variability in the lower limb joint angles during fast-paced walking compared to normal walking may result in increased variability in spatiotemporal gait parameters. Consequently, the variability in walking speed and stride length was significantly larger during fast-paced walking compared with those during comfortable-paced walking (see Table 2). This finding aligns with previous studies indicating that higher gait variability is associated with walking speed (Jordan et al., 2007; Johansson et al., 2016; Padulo et al., 2023). Studies have shown that older individuals prone to falls exhibit significant variability in joint angles and/or gait parameters during gait (Hausdorff et al., 1997; Hausdorff et al., 2001; Brach et al., 2005; Brach et al., 2007; Kobayashi et al., 2014; Johansson et al., 2016; Tsuchida et al., 2022; Inai et al., 2023), and have a higher risk of falling when walking at a faster pace (Quach et al., 2011). Therefore, walking faster than a comfortable pace can increase gait variability, possibly due to greater variability in lower-limb joint angles in the sagittal plane, thereby raising the fall risk in older adults.

Smaller variability, particularly in the knee and ankle joints in the sagittal plane, was observed when emphasizing the joint angle characteristics of the knee sleeve condition. Previous studies have reported that knee joint movement during walking affects ankle joint motion (Sotelo et al., 2018; Attias et al., 2019). This suggests that the reduced variability in the knee joint due to wearing a knee sleeve covering the periphery of the knee joint may have also resulted in decreased variability in the ankle joint. The PCL results revealed that this decrease was most significant during the mid-stance and early to mid-swing phases in the knee joint, as well as during the early swing phase in the ankle joint. These findings highlight the efficacy of knee sleeves in reducing joint variability, particularly in the knee and ankle joints, during the early swing phase. Prior research suggests that in older adults, variability in lower limb joint angles during the swing phase can influence changes in minimum toe clearance, potentially increasing the risk of trip-related falls (Mills et al., 2008; Barrett et al., 2010). In addition, it has been reported that there is greater variability in minimum toe clearance in elderly individuals, particularly those who have a history of falls (Khandoker et al., 2008; Barrett et al., 2010). Therefore, knee sleeves may offer a viable approach to reduce the variability in knee and ankle joint angles during walking, thereby potentially reducing the risk of trip-related falls.

Although the underlying biomechanical mechanisms remain to be elucidated, previous studies have reported an enhanced joint position sense with the use of knee sleeves (Birmingham et al., 1998; Herrington et al., 2005; Mohd Sharif et al., 2017; Dzidotor et al., 2024), elastic bandages (Perlau et al., 1995), and knee braces (McNair et al., 1996). Additionally, knee sleeves have been reported to improve proprioception and muscle co-contraction during walking (Collins et al., 2011). Heightened sensory input from tactile stimulation due to compression may be a key factor in producing these effects (Mohd Sharif et al., 2017; Dzidotor et al., 2024). Therefore, knee sleeves may induce proprioceptive changes, subsequently modulating gait dynamics and stabilizing joint motion, thereby reducing unnecessary joint variability during walking.

While the variability of the lower limb joint angles decreased with the use of knee sleeves, no significant difference was observed in the variability of gait spatiotemporal parameters (Table 2). This could be because we tested healthy older adults, and the effect of knee sleeves may be more pronounced in older adults with functional limitations, such as those with a history of falls and/or frailty. Previous research has demonstrated that variability in the lower limb joints occurs among elderly individuals who have experienced falls or are frail, further affecting the overall gait variability (Kobayashi et al., 2014; Tsuchida et al., 2022). Additionally, our findings indicate that when older people walk at a fast speed, variability in the lower limb joints may lead to increased gait variability. Therefore, knee sleeves may reduce gait variability, particularly in older individuals with a higher risk of falls and/or frailty. Taken together, knee sleeves may offer a valuable method for improving knee and ankle joint angle variability and reducing fall risk.

The present study has several limitations that should be noted. First, soft tissue and knee sleeve artifacts might have introduced bias in the observed plane angles, particularly in the knee joints. Despite placing markers on the bony landmarks of the body, the presence of such artifacts should be considered. Second, the participants in this study were asymptomatic older adults. Hence, these findings may not be applicable to specific patient cohorts or target groups. As the effect of knee sleeves on gait variability in other populations, such as those who have experienced falls or are frail, remains unknown, further research is required to investigate their effects across various demographics.

## 5 Conclusion

PCA revealed that wearing knee sleeves and walking speed influenced several PCVs of the lower limb joint kinematics during gait. We reconstructed the joint kinematics using these vectors and observed larger variability in the hip, knee, and ankle joint angles on the sagittal plane when walking at a faster pace as compared to that at a normal speed and reduced variability in the knee and ankle joint angles on the sagittal plane when walking with knee sleeves compared to those without. These findings suggest that walking faster could increase the variability of the lower limb joint angles, while knee sleeves could decrease the variability in the knee and ankle joint angles. This indicates that knee sleeves could be a valuable tool to reduce the risk of falls by enhancing gait variability in older adults.

## Data Availability

The datasets presented in this article are not readily available because our IRB approval does not include data sharing. Requests to access the datasets should be directed to WT, w-tsuchida@aist.go.jp.
