# Supplementary material for: Effect of knee sleeves on joint angle variability during gait in older adults: a principal component analysis
Source: Front Bioeng Biotechnol. 2025 May 20;13:1525174. doi: 10.3389/fbioe.2025.1525174 (PMC12129960; doi:10.3389/fbioe.2025.1525174)
Supplement: Supplementary file 1 [file DataSheet1.docx]

Supplementary Figure


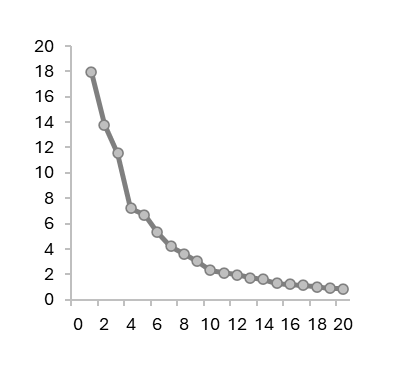


Explained variance

(%)

Principal component

**Supplementary Figure 1**. Scree plot of principal component analysis (PCA), showing the variance explained by each principal component.
